# Ultrastructure of Rat Rostral Nucleus of the Solitary Tract Terminals in the Parabrachial Nucleus and Medullary Reticular Formation

**DOI:** 10.3389/fncel.2022.858617

**Published:** 2022-03-17

**Authors:** Sook Kyung Park, Yi Sul Cho, Jong Ho Kim, Yun Sook Kim, Yong Chul Bae

**Affiliations:** Department of Anatomy and Neurobiology, School of Dentistry, Kyungpook National University, Daegu, South Korea

**Keywords:** gustatory, rNST neurons, synapse, central connectivity, ultrastructure

## Abstract

Neurons in the rostral nucleus of the solitary tract (rNST) receive taste information from the tongue and relay it mainly to the parabrachial nucleus (PBN) and the medullary reticular formation (RF) through two functionally different neural circuits. To help understand how the information from the rNST neurons is transmitted within these brainstem relay nuclei in the taste pathway, we examined the terminals of the rNST neurons in the PBN and RF by use of anterograde horseradish peroxidase (HRP) labeling, postembedding immunogold staining for glutamate, serial section electron microscopy, and quantitative analysis. Most of the anterogradely labeled, glutamate-immunopositive axon terminals made a synaptic contact with only a single postsynaptic element in PBN and RF, suggesting that the sensory information from rNST neurons, at the individual terminal level, is not passed to multiple target cells. Labeled terminals were usually presynaptic to distal dendritic shafts in both target nuclei. However, the frequency of labeled terminals that contacted dendritic spines was significantly higher in the PBN than in the RF, and the frequency of labeled terminals that contacted somata or proximal dendrites was significantly higher in the RF than in the PBN. Labeled terminals receiving axoaxonic synapses, which are a morphological substrate for presynaptic modulation frequently found in primary sensory afferents, were not observed. These findings suggest that the sensory information from rNST neurons is processed in a relatively simple manner in both PBN and RF, but in a distinctly different manner in the PBN as opposed to the RF.

## Introduction

Information on the central connectivity of axon terminals of specific neurons can help understand how the neuronal signals are transmitted and processed by the targeted brain areas. We and others have shown that the pattern of central connectivity of primary sensory afferents differs among individual fiber types (Alvarez et al., 1992, 1993; Bae et al., 2005a; Yeo et al., 2010; Kim et al., 2014). The central connectivity of the primary sensory afferents of a single type also differs with respect to their target nucleus in which they are associated with functionally different neural circuits (Alvarez, 1998). For example, the connectivity of the terminals of a single Aβ low-threshold mechanoreceptive afferent in the trigeminal principal nucleus is different from that found in the trigeminal oral nucleus. These differences may relate to the fact that the principal nucleus is involved in the discriminative aspect of orofacial mechanosensation, while the oral nucleus is involved in reflexive jaw movement (Bae et al., 1994, 2000).

Neurons in the rostral nucleus of the solitary tract (rNST) relay gustatory information from the tongue to the parabrachial nucleus (PBN) and the medullary reticular formation (RF). These two second-order relay nuclei in the taste pathways are involved in the discriminative aspect of taste perception and reflexive orofacial movements, respectively (Travers and Hu, 2000; King, 2006; Zaidi et al., 2008; Jarvie et al., 2021). Electrophysiological studies and immunohistochemical studies with retrograde neural tracing have shown that rNST neurons that project to PBN and RF are mainly glutamatergic (Gill et al., 1999; Nasse et al., 2008).

Recently, we showed that the terminals of chorda tympani afferents, primarily an Aδ fiber, convey gustatory information from the anterior part of the tongue and make simple synaptic connections in the first relay nucleus, the rNST. This organization differs from that of the complex synaptic connections made by non-gustatory Aδ fibers in the trigeminal nuclei (Alvarez et al., 1992; Park et al., 2016). However, less is yet known about the central connectivity of the glutamatergic rNST neurons in the PBN and RF. More detailed study of these structures may help us understand how sensory information from rNST neurons is processed at these secondary relay nuclei in the taste pathway.

To address this issue, we investigated the synaptic connectivity of rNST neurons in the PBN and RF by anterograde tracing with horseradish peroxidase (HRP), postembedding immunogold staining for glutamate, serial section electron microscopy, and quantitative analysis.

## Materials and Methods

### Anterograde Labeling of Axon Terminals of the Rostral Nucleus of the Solitary Tract Neurons

All animal procedures were performed according to the National Institute of Health guidelines and were approved by the Kyungpook National University Intramural Animal Care and Use Committee. Seven 9–10-week-old male Sprague-Dawley rats weighing 300–320 g were used for this study. Animals were anesthetized with an i.p. injection of a mixture of ketamine (40 mg/kg) and xylazine (4 mg/kg) and fixed on a stereotaxic frame (Narishige, Tokyo, Japan). A glass micropipette (internal tip diameter, 20–30 μm) was filled with HRP (5% HRP in 5% DMSO, TOYOBO Co., Osaka, Japan) and inserted stereotaxically into rNST (AP: 2.6–2.65 mm caudal to the interaural line, L: 1.7 mm to the midline, and H: 8.0 mm below the bone surface; Paxinos and Watson, 2005). The tracer was injected iontophoretically (1.2–2 μA, 7 s on/off cycle, for 8 min), and the micropipette was kept in position for another 6 min after the injection.

### Tissue Preparation

After 24–27 h, the rats were deeply anesthetized with a mixture of ketamine (80 mg/kg) and xylazine (10 mg/kg, i.p.) and were perfused transcardially with freshly prepared fixative containing 2.5% glutaraldehyde, 1% paraformaldehyde, and 0.1% picric acid in 0.1 M phosphate buffer (PB, pH 7.4). The brainstem was removed and postfixed in the same fixative for 2 h at 4°C. Then, sections were cut coronally on a vibratome at 60 μm. Anterogradely transported HRP was revealed using a tungstate/tetramethylbenzidine protocol (Weinberg and van Eyck, 1991) and stabilized with diaminobenzidine in PB (pH 6.0). Wet sections were examined on a light microscope, and further studies were performed only on the material in which the tracer was concentrated in the rNST, including its rostral central subdivision. Sections of three rats showing that HRP is confined to rNST, among seven rats that HRP was injected iontophoretically, were used for electron microscopy. Sections containing HRP-labeled puncta in the PBN and RF were selected, postfixed in 0.5% osmium tetroxide in PB for 25 min, dehydrated in the graded alcohols, flat-embedded in Durcupan ACM (Fluka, Buchs, Switzerland) between strips of Aclar plastic film (EMS, Hatfield, PA, United States), and cured for 48 h at 60°C. Chips containing numerous HRP-labeled puncta in the central medial subdivision of the PBN or in the RF were cut and glued onto blank resin with cyanoacrylate. Serial thin sections were collected on formvar-coated single slot nickel grids. Grids were stained with uranyl acetate and lead citrate and examined on a Hitachi H-7500 electron microscope (Hitachi, Tokyo, Japan) at 80 kV accelerating voltage. Electron micrographs at a final magnification of 30,000× were taken from every other section through the HRP-labeled boutons with a Digital Micrograph software driving a cooled CCD camera (SC1000; Orius; Gatan, Pleasanton, CA, United States), attached to the microscope, and saved as TIFF files. The brightness and contrast of the images were adjusted using Adobe Photoshop CS5.1 (Adobe Systems Inc., San Jose, CA, United States).

### Postembedding Immunogold Staining for Glutamate

One in every 5–6 grids containing serial thin sections were selected for immunogold staining for glutamate, which was performed as described previously from our laboratory (Park et al., 2019; Paik et al., 2021). In brief, grids were treated in 1% periodic acid for 6 min to etch the resin, followed by 9% sodium periodate for 10 min to remove the osmium tetroxide, and then washed in distilled water. After incubation in 2% human serum albumin (HSA) in Tris-buffered saline containing 0.1% Triton X-100 (TBST; pH 7.4) for 10 min, the grids were incubated with rabbit antiserum against glutamate (1:30,000; G6642; Sigma-Aldrich, St. Louis, MO, United States) in TBST containing 2% HSA for 3.5 h at room temperature. To eliminate cross-reactivity, the diluted antiserum was preadsorbed with glutaraldehyde (G)-conjugated amino acids, namely, 300 μM glutamine-G, 100 μM aspartate-G, and 200 μM β-alanine-G. After rinsing in TBST, the grids were incubated for 1.5 h in goat anti-rabbit IgG coupled to 15 nm gold particles (1:25 in TBST containing 0.05% polyethylene glycol; BioCell Co., Cardiff, United Kingdom). After washing in distilled water, the grids were counterstained with uranyl acetate and lead citrate and then examined on a Hitachi H-7500 electron microscope at 80 kV accelerating voltage. To assess the immunoreactivity for glutamate, the gold particle density of HRP-labeled boutons was compared with the average tissue density in 10–15 randomly selected areas (2 μm^2^ each, a total area of 20–30 μm^2^ per section). Boutons containing gold particles at a density greater than the average tissue density + 2.576 SD (significant difference at 99% confidence level) were considered glutamate-immunopositive (Paik et al., 2019, 2021); immunogold labeling over mitochondrial profiles was excluded from the analysis. Measurements were performed on electron micrographs using a digitizing tablet and Image J software. We analyzed synaptic connectivity of 50 and 43 labeled boutons that are Glut+ in the PBN and RF, respectively, from three rats (15–18 boutons per rat in the PBN and 13–16 boutons per rat in the RF).

The specificity of the glutamate antiserum was confirmed on test thin sections of “sandwiches” of rat brain macromolecule-glutaraldehyde fixed complexes of different amino acids, such as GABA, glutamate, taurine, glycine, aspartate, and glutamine (Ottersen, 1987; Bae et al., 2000). Test sections were also incubated in the same drops of glutamate antiserum as the tissue sections, and the respective conjugate in the test sections was selectively labeled. Omission or replacement of the primary antisera with normal goat serum abolished the immunostaining. Consistency of immunostaining was also confirmed in consecutive thin sections of the same boutons.

## Results

**FIGURE 1:**
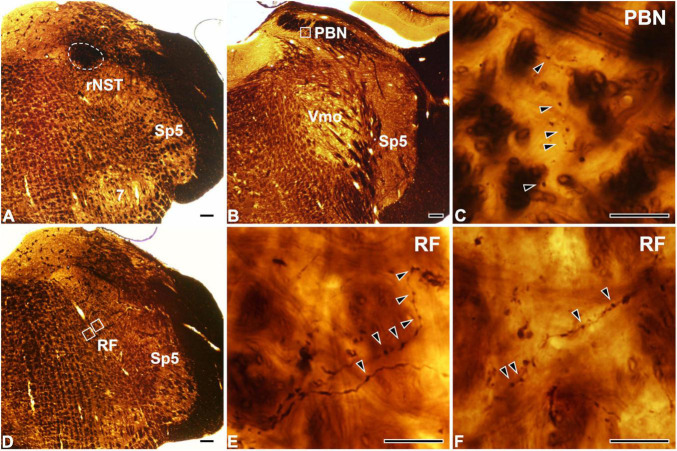
Light micrographs showing the tracer injection site in the rostral part of the nucleus of the solitary tract (rNST; **A**) and anterogradely labeled axon terminals in the parabrachial nucleus (PBN; **B,C**) and the reticular formation (RF; **D–F**). **(A)** The horseradish peroxidase (HRP) injection, outlined with a dashed line, is confined to the rNST. **(B–F)** Dark HRP-labeled axons and terminals (arrowheads) are observed in the central medial subnucleus of the PBN **(B,C)** and the medullary RF **(D–F)**. Panel **(C)** is the enlargement of the square in panel **(B)**. Panels **(E,F)** are enlargements of the medial and lateral squares, respectively, in panel **(D)**. Sp5, spinal trigeminal nucleus; Vmo, trigeminal motor nucleus; 7, facial nucleus. Scale bars, 200 μm in panels **(A,B,D)** and 20 μm in panels **(C,E,F)**.

**FIGURE 2:**
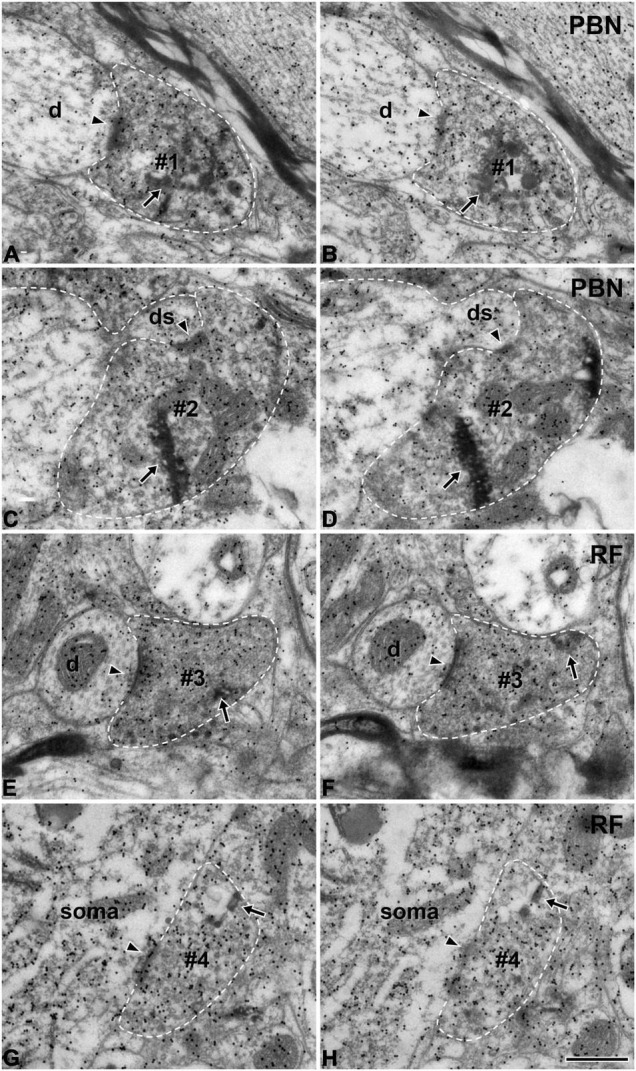
Electron micrographs of immunogold staining for glutamate in adjacent thin sections through anterogradely labeled Glut+ terminals (#1–#4) in the parabrachial nucleus (PBN, **A–D**) and medullary reticular formation (RF, **E–H**) after tracer injection in the rNST. Postsynaptic targets of Glut+ labeled terminals (boutons) are different in the PBN and RF. Glut+ labeled terminals are presynaptic to the dendritic spine more frequently in the PBN than in the RF, while they are presynaptic to soma or proximal dendrite more frequently in the RF than in the PBN. **(A–D)** The labeled terminals (#1, #2) in the PBN establish synaptic contacts (arrowheads) with a dendritic shaft (d; **A,B**) and a dendritic spine (ds; **C,D**). **(E–H)** The labeled terminals in the RF (#3, #4) establish synaptic contacts (arrowheads) with a small dendritic shaft (d; **E,F**) and a cell body (soma; **G,H**). The labeled terminals can be identified by the presence of the HRP reaction product (arrows) within the axoplasm. Labeled terminals that are Glut+ were analyzed in this study: note the high density of gold particles coding for glutamate over the areas of the axoplasm containing synaptic vesicles. Glut immunoreactivity is consistent in the pairs of serial sections of the labeled terminal, confirming their glutamatergic nature. The labeled terminals and the postsynaptic dendritic spine are outlined with a dashed line for clarity. Scale bar, 500 nm.

At the light microscopic level, the injected HRP was confined to the rNST (Figure 1A). Dark HRP-labeled puncta, which is how labeled axon terminals appear at that level of magnification, were dense in the medial PBN (Figures 1B,C) and in the medullary RF (Figures 1D–F), ipsilateral to the tracer injection. At the electron microscopic level, HRP-labeled axon terminals (boutons) were identified by the presence of HRP reaction product (arrows, Figure 2), appearing as electron-dense rods or amorphous deposits within the axoplasm. Most labeled boutons had dome or ellipsoid shape, whereas scalloped or glomerular shape, typical of the primary somatosensory afferent terminals, was not observed. In glutamate-immunopositive (Glut+) boutons, the gold particles labeling for glutamate were dense over regions containing synaptic vesicles and over mitochondria (Figure 2): the gold particle density for Glut was 1.2–19.5 times higher than the average tissue density + 2.576 SD. Boutons labeled with anterogradely transported HRP were mostly Glut+ and established synaptic contacts (arrowheads, Figure 2) of asymmetrical type with postsynaptic dendrites or somata. Glut immunoreactivity was consistent in the adjacent serial sections of the labeled boutons, confirming their glutamatergic nature (Figure 2). Most of the profiles postsynaptic to the anterogradely labeled terminals were also Glut+ (Figures 2A–D,G,H), although the number of gold particles over others did not reach the level of Glut+ (Figures 2E,F). By EM observation, postsynaptic targets of Glut+ labeled boutons differed in the PBN compared with the RF, which led to statistical analysis on the serial sections of the Glut+ labeled boutons.

We analyzed 50 anterogradely labeled boutons that were Glut+ in the centromedial nucleus of the PBN and 43 anterogradely labeled boutons that were Glut+ in the parvocellular and intermediate regions of the RF. The large majority of the labeled boutons (94.0% in the PBN and 97.7% in the RF) established a synaptic contact with a single postsynaptic element, and the remaining with two postsynaptic elements (we did not observe any boutons that established synaptic contacts with more than two postsynaptic elements, Table 1). Most postsynaptic elements in both PBN and RF were dendritic shafts. Glut+ labeled boutons in the PBN frequently established synapses with dendritic spines (14.0% vs. 2.3% in the PBN vs. RF, respectively), whereas Glut+ labeled boutons in the RF frequently established synapses with soma or proximal dendrites that contained rough endoplasmic reticulum, suggesting their proximity to the cell body (4.0% vs. 27.9% in the PBN and RF, respectively). The frequency of synaptic contacts between labeled boutons and dendritic spines was significantly higher in the PBN than in the RF, whereas the frequency of synaptic contacts between labeled boutons and soma or proximal dendrites was significantly higher in the RF than in the PBN (Table 2). None of the labeled boutons established axoaxonic synapses.

**TABLE 1:** Frequency (%) of occurrence of terminals of the rostral nucleus of the solitary tract (rNST) neurons in the parabrachial nucleus (PBN) and medullary reticular formation (RF), according to the number of their postsynaptic profiles.

Region	Number of terminals examined	No. of postsynaptic profiles	
		1	2
PBN	50	94.0 (47/50)	6.0 (3/50)
RF	43	97.7 (42/43)	2.3 (1/43)

*n in parentheses indicates the number of terminals/total number of terminals examined.*

**TABLE 2:** Frequency of occurrence (mean ± SD) of different types of synaptic contacts per labeled terminal of rNST neurons in the PBN and medullary RF.

Region	Number of terminals examined	Type of postsynaptic profile			Total number of contacts
		Soma/proximal dendrite	Dendritic shaft	Dendritic spine	
PBN	50	0.04 ± 0.20* (*n* = 2)	0.88 ± 0.44 (*n* = 44)	0.14 ± 0.35* (*n* = 7)	1.06 ± 0.24 (*n* = 53)
RF	43	0.28 ± 0.45* (*n* = 12)	0.72 ± 0.45 (*n* = 31)	0.02 ± 0.15* (*n* = 1)	1.02 ± 0.15 (*n* = 44)

*“n” is the total number of synaptic contacts for all examined boutons.*

*Indicates statistically significant differences between PBN and RF (unpaired t-test, p < 0.05).

## Discussion

The main finding of this study is that most Glut+ terminals from the rNST contact a single dendrite in both PB and RF, suggesting that sensory signals from the rNST are processed in a relatively simple manner in both these nuclei. In contrast, we observed that the frequency with which these labeled terminals established contacts on somata and proximal dendrites vs. dendritic spines differed between the PBN and RF target nuclei. This suggests that there are distinct differences in how the sensory information from the rNST is handled by the PB and RF.

### Rostral Nucleus of the Solitary Tract Afferent Terminals Establish Simple Synaptic Connections in the Parabrachial Nucleus and Reticular Formation

In both PBN and RF, most Glut+ axon terminals of rNST neurons synapsed with a single postsynaptic dendrite or cell body, suggesting that, at the single bouton level, the afferent signal is transmitted to a specific group of target neurons with less synaptic divergence. This type of synaptic connectivity is analogous to that of axon terminals of the second-order neurons receiving primary sensory input and involved in the reflexive jaw movement, such as the neurons in the supratrigeminal nucleus, that receive proprioceptive input from jaw-closing muscle spindle afferents and project to trigeminal motor nucleus (Paik et al., 2009). However, it is very different from the synaptic connectivity of the chorda tympani afferents conveying gustatory input from the anterior tongue to the rNST. In this nucleus, the large majority of their axon terminals contact two or more dendrites (Park et al., 2016). Similarly, the organization of the rNST projections is also simpler than that of the somatosensory primary afferents in the spinal dorsal horn and trigeminal sensory nuclei, where the terminals usually contact multiple (2–13) dendrites (Alvarez et al., 1992; Bae et al., 2003, 2005a; Kim et al., 2008; Yeo et al., 2010; Park et al., 2019). Thus, the axon terminals of second-order neurons that receive gustatory or proprioceptive input from primary sensory afferents contact few postsynaptic neurons in the second-order relay nuclei, in contrast with the axon terminals of primary sensory afferents, which usually contact multiple dendrites in the first-order relay nuclei, suggesting the spread of sensory information to multiple postsynaptic neurons.

In some cases, axoaxonic synapses in the dorsal horn and trigeminal nuclei provide the basis for presynaptic modulation of the primary afferent input and are implicated in the sharpening of the sensory signal (Gray, 1962; Rudomin and Schmidt, 1999). For example, the primary somatosensory afferents, including Aβ and Aδ afferents, frequently receive axoaxonic synapses from GABAergic terminals (Alvarez et al., 1992; Bae et al., 2005b; Moon et al., 2008; Park et al., 2019). Chorda tympani afferents also frequently receive axoaxonic synapses from GABAergic axon terminals in the rNST, suggesting presynaptic modulation of the gustatory signal before it is relayed to the postsynaptic neurons in the first-order relay nuclei (Park et al., 2016). In this study, however, none of the rNST afferents in PBN and RF were involved in axoaxonic synapses, suggesting that, in contrast to the primary sensory afferents, rNST neurons relay taste information to their postsynaptic neurons without presynaptic modulation.

### Afferents of Rostral Nucleus of the Solitary Tract Neurons Have Different Somatodendritic Targets in the Parabrachial Nucleus and Reticular Formation

The spatial distribution of excitatory input along the soma-dendritic tree of the postsynaptic neuron plays a role in determining its influence, so that the input onto the soma/proximal dendrite compartment exerts a more powerful effect on the excitability of the postsynaptic neurons, and that onto the distal dendrites exerts a weaker effect (Burke, 2004). The frequency of labeled boutons of rNST axons establishing synapses within the soma/proximal dendrite was seven times higher in the RF, a center for reflexive orofacial motor control, than in the PBN, a center for taste perception (Bradley and Kim, 2006; King, 2006). This arrangement is analogous to the primary somatosensory afferents that show more frequent contact with the soma/proximal dendrite compartment of the postsynaptic neurons in the trigeminal oral nucleus, which controls reflexive oral motor behavior, than in the trigeminal principal nucleus, which is involved in the somatosensory perception (Bae et al., 2000). Axon terminals of premotor neurons also very frequently form synapses with the soma/proximal dendrite compartment of the postsynaptic neurons in the trigeminal motor nucleus (Shigenaga et al., 2000; Paik et al., 2009). All of the above suggests that the terminals of excitatory neurons that mediate motor reflexes may exert a stronger influence on the activity of the postsynaptic neurons than those that mediate sensory perception.

We observed far more contacts between rNST terminals and spines in the PBN. Dendritic spines undergo activity-dependent plasticity with respect to their profile diameter, number, and the size of their postsynaptic densities, all resulting in changes in the strength of the synaptic transmission (Harms and Dunaevsky, 2007; Baczynska et al., 2021). Many studies have shown that synaptic plasticity is common in the brain regions involved in gustatory perception (Hill, 2004; Dekkers et al., 2021; Martin et al., 2021). rNST afferent terminals established synaptic contacts with dendritic spines much more frequently in the PBN than in the RF, and the number of dendritic spines receiving synaptic contact from an rNST afferent terminal was six times higher in the PBN than in the RF. Thus, the plasticity of dendritic spines and the changes in the postsynaptic neuron excitability due to alteration of the input from rNST neurons under pathological or experimental conditions, such as sodium restriction or gustatory nerve section, may be more extensive in the PBN than in the RF.

Many postsynaptic dendrites of the Glut+ labeled boutons in the PBN and RF showed dense gold particles for glutamate, suggesting that they are Glut+, whereas some postsynaptic dendrites showed few gold particles for glutamate, suggesting Glut-immunonegative. Considering that GAD+ or tyrosine hydroxylase+ neurons exist in the PBN and RF (Guthmann et al., 1998; Weihe et al., 2006; Travers et al., 2007), the Glut-immunonegative postsynaptic neurons might be either GABAergic or dopaminergic neurons. Activation of various types of postsynaptic neurons by Glut+ labeled boutons of rNST neurons in the PBN and RF may be involved in the precise modulation of taste perception and reflexive jaw movement.

In summary, our findings suggest that sensory information from rNST neurons in the second-order relay nuclei of taste pathways is processed in a distinct manner that is different from gustatory information from the tongue in the first relay nucleus, rNST, and that it is processed differently between in the PBN and in the RF.

## Data Availability Statement

The original contributions presented in the study are included in the article/supplementary material, further inquiries can be directed to the corresponding author.

## Ethics Statement

The animal study was reviewed and approved by Research and Ethics Committee of Kyungpook National University.

## Author Contributions

YB designed the study. SP, YC, and JK contributed to tracing, postembedding staining, and electron microscopy. SP and YB wrote the manuscript. All authors contributed to analysis and interpretation of the data and have full access to all the data in this study and take responsibility for the integrity of the data and the accuracy of the data analysis.

## Conflict of Interest

The authors declare that the research was conducted in the absence of any commercial or financial relationships that could be construed as a potential conflict of interest.

## Publisher’s Note

All claims expressed in this article are solely those of the authors and do not necessarily represent those of their affiliated organizations, or those of the publisher, the editors and the reviewers. Any product that may be evaluated in this article, or claim that may be made by its manufacturer, is not guaranteed or endorsed by the publisher.
